# The Pacified Face: Early Embodiment Processes and the Use of Dummies

**DOI:** 10.3389/fpsyg.2020.00387

**Published:** 2020-03-13

**Authors:** Magdalena Rychlowska, Ross Vanderwert

**Affiliations:** ^1^School of Psychology, Queen’s University Belfast, Belfast, United Kingdom; ^2^School of Psychology, Cardiff University, Cardiff, United Kingdom

**Keywords:** pacifier, emotion, embodiment, sensorimotor simulation, facial mimicry

## Abstract

Few things affect us as much as facial expressions, as they inform us about others’ feelings and intentions, thereby influencing our own emotions and behaviors. A substantial body of literature links the critical abilities of recognizing and understanding emotion displays with facial mimicry, a sensorimotor process involving rapid imitation of perceived expressions. For example, blocking or altering facial mimicry in adults leads to disruptions in judgments in emotion recognition or emotional language processing. The present review focuses on pacifier use in infancy, a common practice that has the potential to interfere with infants’ facial movements in ways identical to laboratory paradigms designed to block facial mimicry. Despite this similarity and the widespread use of infant soothers, little is known about their long-term effects. Here we review studies exploring the psychological correlates and implications of pacifier use. In particular, we discuss how soothers may interfere with the development of social skills in infants and present evidence linking pacifier use with disrupted adults’ mimicry of facial expressions displayed by infants. Other preliminary findings reveal negative correlations between the use of soothers and children’s spontaneous facial mimicry as well as emotional competence of young adults. Such studies, although correlational, suggest that this widespread parenting practice may affect the development of social skills by influencing emotional coordination. We discuss the implications of these findings and propose avenues for future research that can provide insights into the role of embodied processes in the development of emotional competence and adult functioning.

## Introduction

The use of pacifiers, or dummies in the United Kingdom, is one of the most ancient and widespread parenting methods. The practice is at least 3,000 years old and predecessors of today’s silicone pacifiers include items such as sugar rags or feeding dummies made of clay, silver, pearl, or coral that have been discovered in excavations or depicted in art (Levin, 1971). Although statistics of pacifier use vary widely across the globe (see Flam, 2014, for review), a study of a cohort of American mothers reveal that 68% of them introduced a pacifier before 6 weeks (Howard et al., 1999), and the rates seem to be similar in Canada (Kramer et al., 2001). Despite the popularity of pacifiers and their efficiency in regulating negative emotion (e.g. Woodson et al., 1985), surprisingly little research has investigated how long-term pacifier use, inducing repeated restriction, or alteration of facial movements, affects infants’ psychological characteristics. The present review focuses on how pacifiers may influence the development of social competences relying on facial expressions and emotion communication.

The article is divided in six sections. The first section describes the central claims of embodiment theories of emotion. The second provides a brief overview of existing research on pacifier use and discusses the relevance of this practice for understanding the role of imitation and embodied processes in emotion processing. The next three sections present findings of previous studies linking pacifiers with reductions of spontaneous facial mimicry in children, lower levels of young adults’ emotional competence, and decreased adults’ facial reactions to infants’ emotion expressions. The last section outlines the key questions relating to the use of pacifiers and directions for future research.

## Embodied Simulation and Emotion Recognition

Theories of embodied or sensorimotor simulation propose that people use their bodies to understand others’ emotions and experiences. According to these accounts (e.g. Goldman and Sripada, 2005; Pitcher et al., 2008; Wood et al., 2016; Ferrari and Coudé, 2018), seeing facial expressions and gestures triggers an active representation of others’ feeling states generated in the motor, somatosensory, and reward brain circuits. This representation serves as a basis for emotion recognition and may even shape visual perception (Wood et al., 2015; Lomoriello et al., 2019). It is worth noting that sensorimotor simulation is only one of the multiple ways through which people can extract information from others’ bodies and faces. Judgments of meanings of facial and bodily expressions can be guided by perceptual expertise, where observers compare diagnostic features of a given expression to their stored representations of different emotion categories (Smith et al., 2005; Folstein et al., 2012). Moreover, interpretation of others’ displays is often informed by the context in which a given expression occurs, as characteristics of the expresser, perceiver, and the situation provide cues about people’s emotions and behavioral intentions (van Kleef et al., 2016; Hess and Hareli, 2017; Greenaway et al., 2018). Compared to these processes, sensorimotor simulation of emotion displays is predicted to partially or fully reactivate affective states and bodily changes associated with experiencing a given feeling. This more complex route to emotion recognition is predicted to be especially important when the emotion expression is subtle or ambiguous, when there is limited contextual information, and when the perceiver is motivated to actually decode the intentions of the expresser (Niedenthal et al., 2010; Beffara et al., 2012).

Sensorimotor simulation is closely related to the concept of facial mimicry, or rapid, automatic, and unconscious imitation of perceived facial expressions (Dimberg and Thunberg, 1998). While facial mimicry represents only part of sensorimotor processes (Wood et al., 2016), a large body of literature links it with recognition of facial expressions of emotion. Existing research used various paradigms, such as testing participants who underwent Botox injections inducing temporary denervation of facial muscles involved in frowning (Hennenlotter et al., 2008; Havas et al., 2010; Wollmer et al., 2012); studying patients with facial paralysis (e.g. Bogart and Matsumoto, 2010); measuring the activity of facial muscles (e.g. Hess and Blairy, 2001); or blocking mimicry (e.g. Oberman et al., 2007; Ponari et al., 2012). The last option involves various alterations of participants’ facial movements, ranging from stickers on subjects’ foreheads (Ponari et al., 2012) to rugby mouthguards (Rychlowska et al., 2014a).

One of the most common methods of inhibiting facial activity in the lower face involves the so-called pen-in-mouth procedures, where participants are asked to hold a pen in their mouth (e.g. Niedenthal et al., 2001; Soussignan, 2002; Oberman et al., 2007; Maringer et al., 2011; Ponari et al., 2012; Kosonogov et al., 2015; Kuehne et al., 2019). Oberman et al. (2007) asked participants to place a pen horizontally in their mouth and hold it in their teeth while not allowing their lips to touch the pen. This “bite” manipulation was shown to consistently increase the activity of four facial muscles and, in a subsequent emotion recognition task, impaired participants’ ability to correctly label facial expressions of happiness. This result suggests that interfering with specific groups of facial muscles leads to selective deficits in the recognition of emotion expressions that engage these muscles. Ponari et al. (2012) later extended these findings by selectively blocking contractions of lower and upper parts of participants’ faces. The “lower” manipulation was equivalent to the “bite” procedure described earlier and consisted of participants holding a chopstick in their mouth horizontally and exerting a constant pressure with the teeth, without allowing their lips to touch the chopstick. In the upper face manipulation condition, participants were asked to draw together two small stickers placed on the inner side of their eyebrows. Compared to the control condition, in which participants could freely move their face, the upper face manipulation made participants less accurate in categorizing facial expressions of anger, and the lower face condition decreased the recognition accuracy of happiness and disgust. Both manipulations negatively affected the labeling of fear but did not seem to influence participants’ ability to recognize surprise and sadness.

Maringer et al. (2011) used the “pen” technique to block participants’ facial mimicry. Half the subjects were asked to hold a pen sideways between their lips and teeth without exerting pressure. The other group of participants could freely move their face. Both groups watched animated sequences of true and false smiles and evaluated the genuineness of those expressions. Participants whose facial muscles were unrestricted could easily distinguish between the two smile types. However, this ability was impaired in participants whose facial muscles were busy with the pen-in-mouth manipulation. In other words, these subjects did not see any difference between fake and genuine smiles. A subsequent study, in which participants saw only genuine smiles, revealed that people whose facial mimicry was blocked relied on contextual information when interpreting these smiles. Specifically, smiles presented in a positive context and supposedly displayed by a salesclerk who had just sold a pair of shoes were interpreted as more genuine than those presented in an ambiguous context, supposedly displayed by a salesclerk trying to sell a pair of shoes. However, participants who could freely move their faces were not influenced by context and perceived the smiles as equally genuine. Taken together, these results implicate activity of specific facial muscles in the recognition of the facial expressions in which those muscles are involved.

It is worth noting that the effects linking facial movements with the recognition of facial expression in adults tend to be small in size (Coles et al., 2019) and depend on other factors (Hess and Fischer, 2013). In particular, studies in which facial mimicry of perceived expressions was measured rather than blocked (e.g. Hess and Blairy, 2001; Fischer et al., 2012a) did not show associations between facial movements and accuracy of emotion recognition. The same was true for studies in which recognition tasks employed prototypical facial expressions (e.g. Blairy et al., 1999). According to recent theoretical accounts (Niedenthal et al., 2010; Hess and Fischer, 2013), these seemingly conflicting findings suggest that sensorimotor processes are preferentially recruited during challenging recognition tasks or when the facial expression is motivationally important for the observer. In addition, facial mimicry is only a part of sensorimotor processes (Wood et al., 2016), and the ability to simulate a movement may be more important than performing it. Such interpretation is supported by evidence that inhibiting the activity of primary motor brain regions, which are involved in generating facial mimicry, disrupts the recognition of smiles to a greater extent than inhibiting the activity of somatosensory brain areas, which are arguably involved in receiving feedback from the face (Korb et al., 2015). Altogether, sensorimotor simulation is a complex and poorly understood phenomenon that needs further exploration given its importance in social learning (e.g. Paulus et al., 2011; de Klerk et al., 2014; Rayson et al., 2017; O’Sullivan et al., 2018; de Klerk et al., 2019), interpersonal bonding (e.g. Meltzoff and Marshall, 2018), body representations (Meltzoff et al., 2018), social cognition (Meltzoff, 1990, 2007), and language processing (e.g. Yeung and Werker, 2013; Bruderer et al., 2015).

Although the effects of facial mimicry tend to be inconsistent in adults (Coles et al., 2019, see also Reisenzein and Stephan, 2013; Wagenmakers et al., 2016; Marsh et al., 2018), their importance and nature may vary during the lifespan. For example, adults with Moebius syndrome, a congenital form of facial paralysis, are able to recognize photographs of emotion expressions with the same accuracy as control participants (Bogart and Matsumoto, 2010). However, a recent study (De Stefani et al., 2019) revealed that, compared with a control group, children with Moebius syndrome were slightly less accurate in labeling videos of facial expressions and showed less pronounced responses of parasympathetic system during observation of social stimuli. In addition, although adults with acute facial palsy are able to accurately recognize facial expression, these judgments can take longer than in control participants (Storbeck et al., 2019). Importantly, no such delay is observed for mere face recognition.

Those and similar results suggest that the effects of disrupting sensorimotor processes may be subtle and differ depending on participants’ age. Thus, investigating these processes from the developmental perspective can shed more light on the role of bodily experience in emotion understanding and social competence. Here, we argue that altering facial mimicry during infancy can be more consequential than in adult age. Early social interactions and free play are critical for child development and existing literature highlights the importance of the first years of life for the development of emotion recognition as well as other social skills (e.g. Nelson, 1987; Stern, 1985a/2000, 2010; Jones, 2007; Hoehl and Striano, 2010; Leppänen and Nelson, 2012; Xie et al., 2018). During this time, faces and face-like patterns are among the most captivating visual objects in babies’ environment (e.g. Farroni et al., 2005). The ability to perform a surprisingly wide range of facial expressions and imitative gestures is present in newborns and emerges as early as in the last trimester of fetal life (e.g. Meltzoff and Moore, 1977, 1989; Nagy, 2011; Reissland et al., 2011, 2013a; Nagy et al., 2014; Delafield-Butt and Trevarthen, 2015). While infant facial and bodily expressions do not necessarily reflect internal states comparable to those experienced by adults, this flexibility of facial and bodily movements is a basis for establishing a repertoire of social behaviors including eye contact, smiles, head orienting, or eyebrow raises (Stern, 1985a/2000). Through imitation, these gestures become part of a complex dynamic system, where infant-elicited behaviors of adults elicit infants’ responses and vice-versa (Stern, 1985b, 2001; Tronick, 1989; Fogel et al., 1992). The timing and coordination of such multimodal interactions are critical for the development of social competence (Tronick, 1989; Stern, 2001, 2010; Beebe et al., 2010). At this stage, mimicry and imitation provide a foundation for understanding of infant’s own movements, mapping them onto bodily experiences and feeling states, and establishing rapport with caregivers (Trevarthen, 2015; Meltzoff and Marshall, 2018; Meltzoff et al., 2018; de Klerk et al., 2019). Thus, while restrictions of facial mimicry in adults may affect sensorimotor simulation and recognition of perceived facial expressions in the on-going task, the consequences may be much more far-reaching for infants. In this case, alterations of facial movements could disrupt not only the infant’s immediate understanding of others’ emotion expressions, but also change the caregivers’ responding and alter the social learning processes grounded in early interactions (Tronick, 1989; Meltzoff, 1990; Fogel et al., 1992).

## What’s Dummy Got to Do With It?

It may be challenging to run adequately powered studies that involve participants with facial paralysis or that manipulate facial mimicry in infants and children. The use of pacifiers overcomes many of these difficulties and provides a convenient tool for studying sensorimotor processes across the lifespan. In addition to being extremely widespread, this practice bears important similarities to the smile-inhibiting paradigm Strack and colleagues employed (Strack et al., 1988), when they asked participants to hold a pen tightly with their lips without touching it with their teeth. Not only can pacifiers engage infants’ facial muscles in a way that inhibits or alters the production of emotion expressions but the plastic shield of a pacifier can hide infants’ expressions from others, disrupting social interactions. In addition, pacifiers are used beyond research laboratories, for extended periods of time, often during the day, and in the presence of caregivers, strangers, or other children. Last but not least, babies are introduced to dummies during early developmental periods, when they learn about the meaning of facial expressions, and when their emotional competence starts to emerge (Stern, 1985a/2000; Campos et al., 2003; Jones, 2007; Pascalis et al., 2011; Leppänen and Nelson, 2012; Ruba et al., 2019). At around 3 months or earlier, infants begin social smiling and show a preference for smiling faces (Kuchuk et al., 1986; Farroni et al., 2007 but see also Reissland et al., 2011), and discrimination between positive and negative emotions emerges between 6 and 9 months (Nelson et al., 1979; Nelson and De Haan, 1996, although see also Reissland et al., 2013b). During these early periods, visual feedback from adults who imitate infants’ facial expressions is predicted to be critical for developing perceptual-motor couplings for facial actions, which underlie the development of spontaneous facial mimicry (Stern, 1985b, 2001, 2010; Ray and Heyes, 2011). Thus, if blocking or altering facial mimicry affects emotion recognition and coordination, the use of pacifiers should be a powerful and ecologically valid paradigm for exploring these effects.

Studying potentially negative consequences of dummy use is also important given the sheer popularity of this practice. As mentioned, most parents in Western countries attempt to introduce pacifiers to their babies (Howard et al., 1999; Kramer et al., 2001) as early as in the first week of life, and the rates remain high at 5 months (Pansy et al., 2008). The decision to introduce pacifiers is mostly motivated by their soothing effects (Woodson et al., 1985; Corbo et al., 2000; Pansy et al., 2008) and convenience: even if other calming methods such as breastfeeding, carrying, or rocking may be more efficient (Kramer et al., 2001), pacifiers can be combined with other methods such as swaddling without actively involving the caregiver (Campos, 1989). Dummies have attracted the medical community’s attention, as they can increase the odds of ear infections (Rovers et al., 2008) and early weaning (Kramer et al., 2001), but they are also associated with reducing the risk of sudden infant death syndrome or SIDS (Hauck et al., 2005). However, research exploring the psychological and social implications of pacifier use is scarce. Among notable exceptions, Gale and Martyn (1996) showed a negative association between the use of dummies and IQ scores in a large cohort of children born in Hertfordshire in the United Kingdom between 1920 and 1930. Although this link remained significant after controlling for social class, the number of older siblings, and the mother’s age, the correlational nature of this finding does not allow to draw causal conclusions. It is also worth noting that Gale and Martyn’s study examined the use of dummies as part of bottle feeding and its results may not generalize to non-nutritive sucking. Finally, the interpretation of the findings is further complicated by evidence linking dummy use with lower socioeconomic status (Gale and Martyn, 1996; Fleming et al., 1999; Mauch et al., 2012; see also Whitmarsh, 2008). Complementing the findings of Gale and Martyn (1996) and Barca et al. (2017) have recently showed an association between prolonged (>3 years) use of pacifiers during social interactions with a child’s difficulties in distinguishing abstract and concrete concepts at the ages of six to seven. In addition, in the study by Lehman et al. (1992), American infants showing a long-term preference for using pacifiers for comfort were less likely to be securely attached to their mothers than infants with a preference for soft objects. Finally, a study conducted by a Brazilian team (Victora et al., 1997) showed that introducing pacifiers was positively associated with rigid, anxious parenting styles, and sensitivity to infant crying. Overall, the preliminary evidence suggesting a link between pacifier use and social cognition lacks an explanatory mechanism. Here we propose that pacifiers may negatively affect emotion competence by altering infants’ facial responding and disrupting coordination during early exchanges between infants and caregivers. Below we present studies exploring this hypothesis.

## Pacifier Use and Spontaneous Facial Mimicry

Niedenthal et al. (2012) were the first to propose that prolonged use of dummies and the associated restrictions in facial mimicry could impact negatively the development of social and emotional skills. The researchers argued that, if pacifiers repeatedly suppress or alter infants’ facial movement, the extended use of a pacifier, especially during interactions between infants and their caregivers, should result in lower levels of spontaneous facial mimicry and the associated emotional competences. The prediction was tested in three studies. In the first study, participants were 7-year olds whose parents provided information on pacifier use and thumb sucking. Although this second behavior is similar to using a pacifier, the researchers predicted it to be less diagnostic of emotional competence, as it is more private and usually controlled by the infant rather than introduced by the parents. Children were filmed while viewing short video sequences representing male and female faces changing from a smile to a sad expression or vice-versa. Two coders who were unaware of the stimulus type or the participants’ pacifier use analyzed video recordings of children’s smiles or sad facial responses during each trial. Counts of these displays, indexing spontaneous facial mimicry, were then analyzed as a function of pacifier use. Results revealed that, although the duration of pacifier use was not significantly associated with children’s facial responses to the videos, there was a significant interaction between sex and the pacifier use. Specifically, the length of using a pacifier was negatively associated with the amount of facial mimicry showed by boys but not girls. Additional analyses examining the effects of pacifier use during the day at home, at night, and during the day outside the home, for example in daycare, and including the frequency of thumb sucking as a control variable, revealed that only the use of dummies during the day at home was associated with lower levels of facial mimicry in children. Here again, the effect was significant only for boys. This result suggests that the use of pacifiers and the related restriction of facial movements are especially impactful when they occur during the day and during the infant’s interactions with the primary caregiver.

## Pacifiers and Emotional Competence of Young Adults

Niedenthal et al. (2012) further argue that, if facial mimicry plays a role in accurate emotion recognition, prolonged restriction of facial movements, and thus, of facial responding, should negatively affect social competences that rely on the accurate recognition of others’ emotions, such as empathy and perspective taking. The two other studies conducted by this research team (Niedenthal et al., 2012) involved large groups of students recruited at French and American universities. Participants completed several scales assessing their social and emotional competence, including the Interpersonal Reactivity Index (IRI; Davis, 1983), which is a standardized scale for measuring empathy, and the Adolescent Short Form of Trait Emotional Intelligence Questionnaire (TEIQue-ASF; Petrides et al., 2006; Mikolajczak et al., 2007). In addition, participants provided information about their use of pacifiers, specifically the ages of onset and offset of pacifier use and thumb sucking, and the frequency with which they used pacifiers in different circumstances. Given that it is possible for young participants not to remember the details of their pacifier use, one of the studies explicitly encouraged subjects to consult the responses with their parents and to check a box on the questionnaire if they had done so. Similarly to the study of 7-year olds reported above, the analysis of the results did not reveal a main effect of pacifier use — this was coded as a binary yes/no variable and as a continuous variable reflecting its length — on participants’ emotional intelligence or ability to understand events from the viewpoint of others, which was measured with the Perspective Taking subscale of the IRI. However, the two studies consistently revealed an interaction of pacifier use with gender, such that pacifier use was associated with lower levels of perspective taking and emotional intelligence in boys but not in girls. These results remained significant when controlling for thumb sucking, mother education, and potentially relevant individual characteristics such as ambivalent attachment (Simpson et al., 1996) and trait anxiety (Gauthier and Bouchard, 1993). Importantly, the findings were not biased by problems in reporting, as they remained the same when participants who had not contacted their parents were excluded from the analyses.

To summarize, these two studies link the use of pacifiers in boys with lower levels of social skills that rely on the accurate identification of others’ expressions. Specifically, the Perspective Taking subscale of the IRI has been linked with the ability to produce specific facial reactions (e.g. Lamm et al., 2008) and to emotion recognition (Morosan et al., 2017). In addition, facets of emotional intelligence (Petrides et al., 2006) involve understanding others’ emotion expressions, recognizing and expressing one’s own feelings, and the ability of adopting someone else’s perspective. Importantly, Niedenthal and colleagues’ three studies (2012) consistently showed that the associations of pacifier use with decreases in these skills were found only in boys and young men. The authors interpret these gender differences in light of the existing literature on sex differences in emotional socialization: the development of emotion competences is generally slower and more fragile in men than in women (Zahn-Waxler et al., 1992; Brody, 2000; Korb et al., 2015). In addition, evidence shows that females tend to recognize emotion expressions more accurately than males (e.g. Babchuk et al., 1985; McClure, 2000; Proverbio et al., 2007; Thompson and Voyer, 2014) and that this advantage is especially marked when integrating visual and auditory stimuli. Women’s higher competence in affect decoding can be an evolutionary adaptation to the role of primary caregiver, where accurate emotion recognition enhances infant survival (Babchuk et al., 1985; Proverbio et al., 2007). While part of women’s advantage in emotion recognition can be ascribed to biology or genetics (de Lacoste et al., 1991; McClure, 2000; Gilmore et al., 2007), cultural factors may also influence this ability. For example, given the social norms dictating that women should be emotion “experts” (Fischer and LaFrance, 2015), parents tend to provide more emotional stimulation to girls, which may compensate for any pacifier-related disruptions in sensorimotor processes (Fivush et al., 2000, but see also Kokkinaki et al., 2019). Alternatively, there may be a broader gender difference in use of facial mimicry for understanding emotions in others exacerbated by pacifier use in infancy. Such interpretation is supported by the results of Wood et al. (2018) recent study, which found that judgments of valence of facial expressions and hand gestures were significantly impaired by restricting facial mimicry in male, but not female adult participants. Further research will be needed to better understand the mechanisms underlying such gender differences in the role of mimicry. These differences should be examined in the light of emotion coordination during early interactions between caregivers and girls versus boys (Weinberg et al., 1999; Cerezo et al., 2017; Kokkinaki et al., 2019). Of particular importance is also the gender composition of mother-infant dyads. Previous research documents greater synchrony during interactions between mothers and sons compared to mothers and daughters (Tronick and Cohn, 1989; Weinberg et al., 1999, but see also Cerezo et al., 2017). Accordingly, between 1.5 and 3 months of life, mothers tend to privilege emotion-related speech during interactions with their sons, rather than daughters, tendency which later changes to attention-related speech (Kokkinaki et al., 2019). How the presence of pacifiers and the resulting disruptions in facial responding affects early intersubjective experiences between same- and cross-gender dyads should be object of future study.

## Pacifiers and Adults’ Facial Reactions to Infants’ Expressions

While pacifiers may influence an infant’s ability to accurately process observed facial expressions and decrease the amount of facial mimicry in children, they may also disrupt an adult’s responding to a baby’s expressions (Niedenthal et al., 2012). Recent research has supported three possible explanations for altered behavior in adults: first, adults may interpret the use of dummies as a sign that the child is difficult and cannot regulate their emotions; second, the shield of a pacifier may hide infants’ emotion expressions; and third, the engagement of facial muscles by the pacifier may disrupt infants’ ability to produce facial expressions. Tellingly, the earliest report of this effect is in the seminal study on neonatal imitation but Meltzoff and Moore (1977). These researchers employed pacifiers during demonstrations of facial gestures in order to prevent changes in the experimenter’s behavior as a result of the infant’s facial expressions.

A subsequent piece of research (Rychlowska et al., 2014b) extended those findings by examining how dummies in babies’ mouths influence adults’ facial reactions to and judgments of infant emotions. The study used photographs of two babies (Gil et al., 2011) displaying expressions of happiness, sadness, and anger, as well as a neutral face. The pictures did not provide contextual information and were edited by the experimenters to create three experimental conditions. In one condition, complete photographs were presented. In the two other conditions, researchers digitally added a pacifier or a white square that covered the baby’s mouth. Female participants watched the stimuli presented on a computer screen and rated the extent to which infants’ faces expressed happiness, sadness, anger, and neutrality. During the task, researchers recorded the electrical activity of participants’ three facial muscles: *zygomaticus major* the main muscle involved in smiling; *depressor labii inferioris*, which lowers the bottom lip in the expression of sadness; and *corrugator supercilii* responsible for frowning and active in the displays of anger and sadness. The analysis of participants’ muscle activity and emotion ratings revealed that, generally, when participants saw the unaltered photographs, they spontaneously frowned in reaction to faces of sad and angry babies and smiled to smiling babies. However, the presence of a pacifier or covering the infant’s mouth by the white square compromised participants’ emotion judgments, such that they perceived less happiness in infant smiling faces and less sadness in infant sad faces. In addition, participants’ zygomaticus major muscle was less active when babies’ smiling faces were covered by the pacifier or the white square rather than presented completely. In other words, obscuring the mouth in the photographs disrupted facial mimicry of infants’ smiles. Those results, and particularly the lack of significant differences between the pacifier and the white square conditions, suggest that covering the infant’s mouth with a pacifier disrupts adults’ imitation of babies’ facial expressions and that those effects are due to hiding perceptual information rather than adults’ negative beliefs about pacifiers. Moreover, the observation that pacifiers disrupted mimicry and recognition of infant smiles rather than angry expressions is consistent with research demonstrating that blocking the mouth region of adult faces selectively impairs recognition of happy facial expressions (Fischer et al., 2012b; Ponari et al., 2012).

Overall, the impaired perception and mimicry of smiles could make interactions with babies using a pacifier less enjoyable and interesting for adults, thus decreasing the amount of social stimulation the babies receive (Stern, 1985a/2000, 2010) and disrupting the mutual feedback system of early emotion exchanges (Fogel et al., 1992; Trevarthen, 2015). In addition, covering the infants’ mouth with a pacifier or with a white square resulted in disrupted judgments of happiness and sadness, emotions that are particularly adaptive for babies in rewarding caregivers (Shore and Heerey, 2011), attracting their attention (Stern, 1985a/2000; Messinger and Fogel, 2007) or soliciting help (Buss and Kiel, 2004). Whatever the mechanism, findings of Rychlowska et al. (2014b) suggest that dummies make adults reduce their facial displays of emotion when interacting with infants. Adults’ facial responses are critical to the development of social mirroring (e.g. Kokkinaki and Kugiumutzakis, 2000; de Klerk et al., 2019) and their reduction occurring in early developmental periods could significantly impair the emergence of emotional skills in infants. This is supported by the evidence that postnatal depression and associated disruptions in mothers’ attunement to their infants (e.g. Levin et al., 1985; Field, 1995; Beebe et al., 2012) have an enduring influence on children’s adjustment and cognitive outcomes (e.g. Field, 1995; Murray et al., 1996, 1999; Field et al., 2009). Importantly, even brief manipulations involving adults’ unresponsive behavior, such as the still-face paradigm simulating maternal depression, lead to significant infants’ distress (e.g. Cohn and Tronick, 1983; Field et al., 1986; Gusella et al., 1988). The consequences of pacifier use are much less dramatic but may still lead to a misregulation of early emotion exchanges by decreasing both adult- and infant-elicited interaction behaviors, thus making free play less enjoyable and rewarding.

## Where Do We Go From Here?

Overall, the studies described provide compelling evidence that pacifier use should be studied in the context of facial expressivity, emotion coordination, and social competence. This is because, first, of the similarity of dummies to standard mimicry-blocking procedures used in the laboratory. Secondly, it is because of the critical importance of early infancy in the emergence of emotion recognition and perspective taking (Stern, 1985a/2000, b, 2001, 2010). Those are the key components of emotional intelligence (Salovey and Mayer, 1990) and are positively associated with life satisfaction (Palmer et al., 2002), social network size (Austin et al., 2005), and health and well-being (Slaski and Cartwright, 2002). Moreover, Gale and Martyn (1996) suggest that pacifiers could influence not only babies’ emotional skills but their general intelligence. By 3 years of age children begin to show reliability in facial expressions, associate them with specific meanings and contexts (Feldman Barrett et al., 2019; Ruba et al., 2019), and be aware of display rules (Cole, 1986). Even beyond these early developmental periods, people’s emotion concepts are affected by the input from their environment (Levari et al., 2018). Plate et al. (2018) recently showed that children and adults adjust their emotion categories based on the frequency of specific emotion expressions that they see. For example, children who saw more calm faces decreased their threshold for categorizing a face as angry. Thus, early emotional experiences, in particular adults’ facial expressions encountered in early childhood, may strongly influence individual differences in emotion perception.

In summary, existing research (Niedenthal et al., 2012; Rychlowska et al., 2014b) reveals negative associations between pacifier use and social competence. By interfering with spontaneous facial mimicry, dummies may disrupt recognition of facial expressions at an age when emotion recognition emerges and is particularly vulnerable to disruption. By altering or inhibiting facial movements over long periods, often during the day and during social interactions with the primary caregiver, pacifiers may also discourage the habit of engaging in spontaneous facial mimicry and impair sensorimotor processing in later life. Moreover, the presence of a dummy can make interactions with babies less interesting and less enjoyable for adults and thus reduce the amount of emotional stimulation that infants receive (Stern, 1985a/2000). Seeing fewer facial expressions or seeing them in different proportions will shape a child’s levels of emotional expressivity or the way in which they form emotion categories. For those reasons, studying the use of pacifiers is a perfect paradigm for examining the role of facial mimicry and other sensorimotor processes in emotion processing. This role has been subject to controversy in recent years (Reisenzein and Stephan, 2013; Coles et al., 2019).

Despite their promising findings, the studies of Niedenthal et al. (2012) and Rychlowska et al. (2014b) provide only indirect support for the involvement of pacifiers in the development of social competences. The former studied facial mimicry and the emotional skills of children and young adults to examine the effects of using a pacifier, which is a behavior that occurred in the distant past (Niedenthal et al., 2012). Thus, findings of these studies heavily rely on parents’ and infants’ memories, which may only partially reflect the actual pacifier use during infancy. Moreover, the resulting evidence, although consistent and grounded in theory, is only correlational, such that reduced facial mimicry and emotional competence in boys could be attributed to factors other than pacifiers, including temperamental difficulties and the fussiness of the infants (e.g. Goldsmith and Harman, 1994; Rothbart, 2007; Radesky et al., 2013), adults’ perceptions of infants’ personality (Bennett, 1971), or individual communication histories (e.g. Lavelli and Fogel, 2002; Hsu and Fogel, 2003). Although Rychlowska et al. (2014b) provided causal evidence for the role of pacifiers and showed that their presence alters emotion perception and facial mimicry in observers, the observers in question were not parents or caregivers but adult women watching photographs of unfamiliar babies in a relatively artificial EMG paradigm (Kamen and Gabriel, 2010). Future research, which would ideally use different methods and include measures of on-going pacifier use, should replicate and extend this initial evidence. It is also necessary to disentangle the effects of pacifiers from individual characteristics of the child and the caregiver, such as infants’ agitation and temperament or caregivers’ personality and mental health. In the past, the use of dummies has been linked to maternal distance, sensitivity to infant crying, and anxious parenting styles (Victora et al., 1997). Studies currently conducted in our laboratory explore the links between pacifier use and parent and infant characteristics, including parental anxiety, socioeconomic status, as well as a child’s personality and fussiness.

Future research should focus on how pacifiers affect emotion processing in babies, as, to date, their influence has only been explored in older children and adults. Ideally, such studies would use multimethod approaches, including neuroimaging, psychophysiology, and visual preference. For example, it is important to test how the presence of pacifier in an infant’s mouth affects early neural processing of facial expressions, in particular those reflecting negative states such as disgust, fear, or anger. This is because accurate discrimination between these expressions emerges later than the understanding of positive displays (Leppänen and Nelson, 2012) and accurate matching of negative emotions to events is a challenging developmental task (Ruba et al., 2019). Another promising avenue is studying how dummies affect infants’ visual preference or selective attention to emotional stimuli. Another critical question that needs to be answered in future studies concerns the facial muscles affected by the pacifiers and the extent to which pacifiers and their different shapes affect facial mimicry. It is also important to examine whether the influences of pacifiers and blocking mimicry in general affect only the processing of facial or visual emotion stimuli or whether cross-modal influences are possible where altered facial movements impair categorization of emotional gestures or sounds (e.g. Bruderer et al., 2015).

Also, what is the role of timing? What is the optimal way of using pacifiers that maximizes their positive effects? Are pacifiers more likely to affect infants’ competences in specific time periods? Such a possibility is hinted to in the study of Barca et al. (2017), in which children who used pacifiers for more than 3 years and during social interactions showed impairments in their ability to discriminate between concrete and abstract concepts. Finally, as mentioned earlier, babies’ responses to facial expressions are only part of a dynamic system involving both the infant and the caregiver (Tronick, 1989; Meltzoff, 1990; Fogel et al., 1992) and, in order to understand the long-term effects of pacifier use it is necessary to examine how dummies influence natural interactions between babies and caregivers. In addition to improving our understanding of the links between pacifiers and emotional competence, such research would shed more light on the soothing effects of pacifiers and their contextual moderators. An ongoing study conducted in our laboratory uses a within-subjects design to examine how the presence of a pacifier in a baby’s mouth influences the exchanges between 12-month-old children and their mothers, in particular measures of facial mimicry, synchrony, and attunement (Kokkinaki et al., 2016). Ideally, longitudinal studies starting before the age of 6 months and examining infants’ responses in laboratory tasks as well as their spontaneous interactions with caregivers and peers would extend our understanding of whether and how pacifier use affects emotional as well as cognitive development. Such research will allow determining which components of early social exchanges are particularly likely to be affected by the use of dummies. While assessing the effects of pacifiers on emotion processing will require a careful triangulation of different methods and subject populations, studying the use of dummies can not only inform recommendations and policies for the optimal use of pacifiers in infants but shed more light on whether and how sensorimotor processes guide emotion recognition and relate to life outcomes. We hope that the present review will attract the attention of psychologists to this promising paradigm.

## Author Contributions

Both authors listed have made a substantial, direct and intellectual contribution to the work, and approved it for publication.

## Conflict of Interest

The authors declare that the research was conducted in the absence of any commercial or financial relationships that could be construed as a potential conflict of interest.
